# Supplementary material for: Phylogenetic Analysis and Characterization of Diguanylate Cyclase and Phosphodiesterase in Planktonic Filamentous Cyanobacterium Arthrospira sp
Source: Int J Mol Sci. 2023 Oct 16;24(20):15210. doi: 10.3390/ijms242015210 (PMC10607523; doi:10.3390/ijms242015210)
Supplement: Supplementary file 1 [file ijms-24-15210-s001.zip › ijms-2657542-Supplementary material S1.pdf]

Supplementary Material

Phylogenetic analysis and characterization of diguanylate cyclase and phosphodiesterase in planktonic filamentous cyanobacterium *Arthrospira* sp.

Kang Wang, [1,2] **Wenjun Li,** [1] **Hongli Cui,** [1] **Song Qin**[1*]

[1] Yantai Institute of Coastal Zone Research, Chinese Academy of Sciences, Yantai 264003, China

[2] University of Chinese Academy of Sciences, Beijing 100049, China

* Corresponding author: Song Qin, email: sqin@yic.ac.cn, Tel: 0086 535 2109005, No. 19, Chunhui Road, Laishan District, Yantai City, Shandong Province, China.

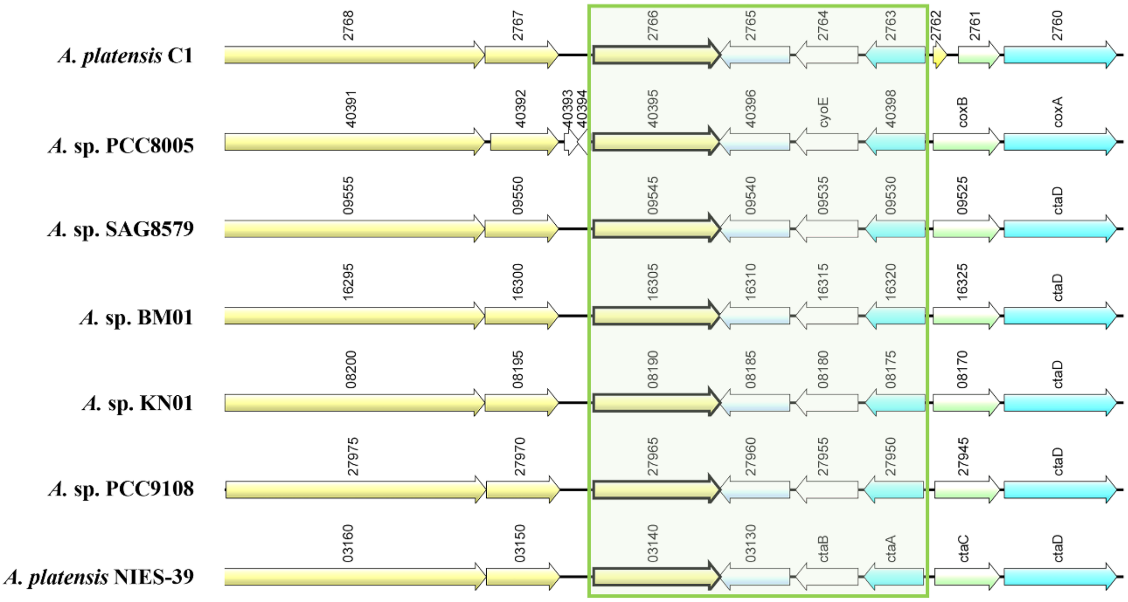

Figure S1. **Synteny analysis of Adc11. Complex syntenies were obtained using the protein sequence of *A. platensis* NIES-39 (D5A515) as a query sequence to search against the indicated *Arthrospira* subspecies.** Consistent color coding permitted the correct identification of both orthologs and paralogs. Genes corresponding to the query proteins are indicated in boldface and boxed with light green.

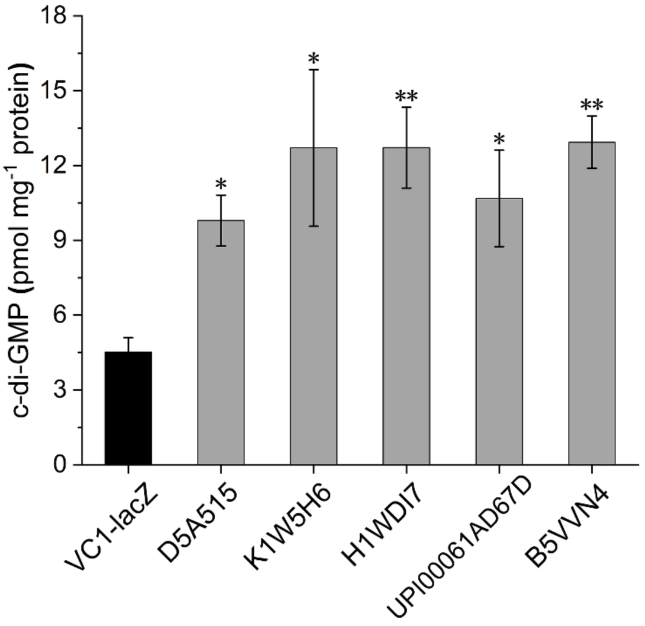

Figure S2. Detection of intracellular c-di-GMP concentration of *E. coli* TOP10 derivatives carrying either the empty vector pBad-VC1-lacZ or the overproducing predicted Adc11 at 24 h in LB medium. The results represent the average ± SD of biological triplicates (n = 3). Asterisks denote the statistical significance of the data according to t-tests with Bonferroni-Dunn correction: *, $p < 0.05$, **, $p < 0.01$.

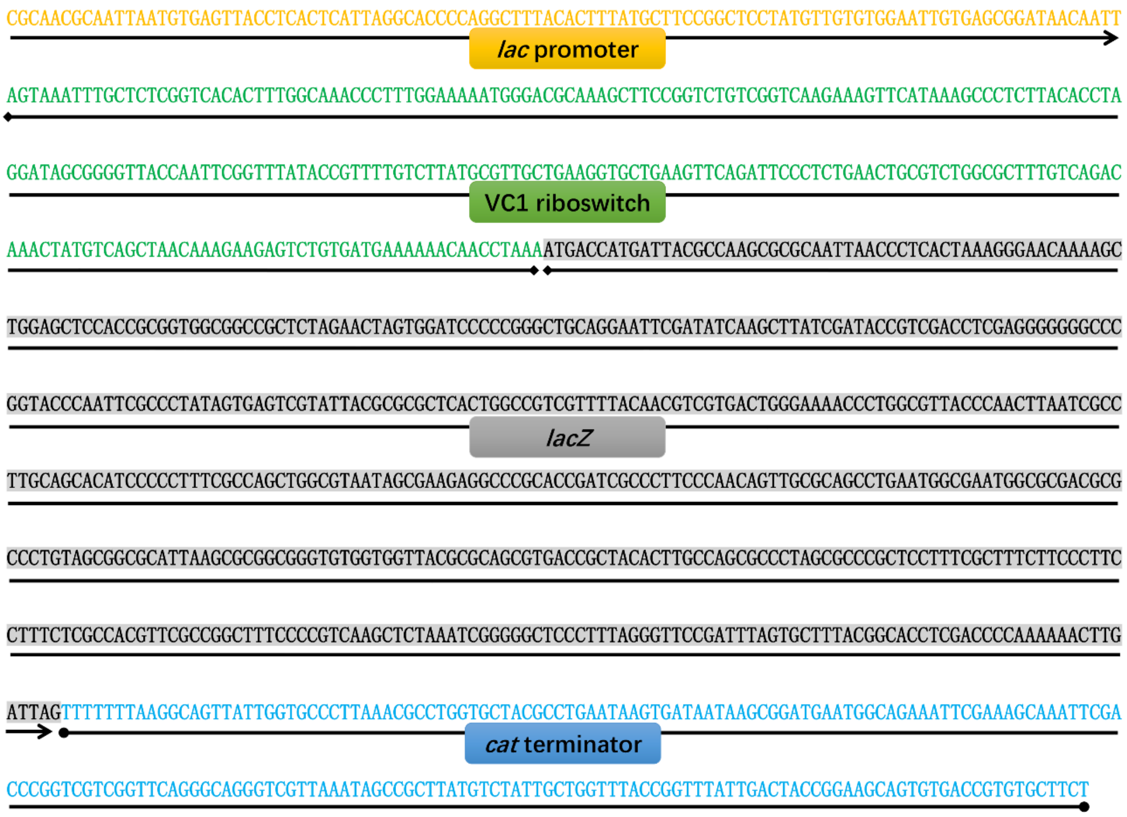

Figure S3. Sequence construction of the *lacZ* expression cassette. The VC1 riboswitch was constructed as the 5' UTR of *lacZ* to controlling *lacZ* expression according to intracellular c-di-GMP concentration.

Table S1. The predicted c-di-GMP proteins in five *Arthrospira* subspecies.

Protein ID in UniProt databases					Conserved motif (variant)	
NIES-39	C1	PCC8005	TJSD091	CS-328	GGDEF	EAL/HDc
D4ZNB7	K1W1Q9	H1WM76	UPI00061A905C		GGEEF	
D4ZP03		H1WL73		B5VYV5	GGEEF	
D4ZT63	K1WT14	H1W802	UPI00061AA153	B5W389	GGDEF	
D4ZTR5					GGEEF	
D4ZX37		H1WHX4		B5W1M1	GGEEF	
D4ZYC7	K1WAY0			B5W6E5	GGEEF	
D4ZZR3	K1WA92	H1WIW0		B5VWD6	GGEEF	
D5A234	K1W441	H1WAS5	UPI00061B2EF1	B5W2T9	GGEEF	
D5A2C5					GGEEF	
D5A2T8					GGEEF	
D5A515	K1W5H6	H1WDI7	UPI00061AD67D	B5VVN4	GGEEF (Adc11)	
D5A338	K1W361	H1W9Z1		B5W3I1	GGEEF	
D4ZXU7					GGEEF	
	K1WBP1	H1WKX2	UPI00061B0BF9	B5VZ91	GGDEF	
				B5W6J6	GGEEF	
D4ZR22	K1XC89	H1WLZ1	UPI00061AD7D0	B5W0E3	GGEEM	
D4ZSS8		H1W9I2		B5VZT6	GGEEM	
D4ZTR2	K1VYW9	H1W8W8	UPI00061AF8BF	B5W7J6	GGEEM	
D5A518	K1W5H9	H1WDI3	UPI0006981A56		GGEEL	
D5A021				B5W0U0	QQAEF	
	K1W3R6	H1WBC0			AGDDC	
				B5W7A8	GGEEI	
D5A4M7		H1WI22		B5VVD7	GGDEF	EAL
D4ZQA1		H1WCY3	UPI00061AD7D3	B5VXY3	GGDEF	EAL
D4ZS72	K1WHE4	H1W7R5	UPI00061B48BD	B5W265	GGDEF	EAL
D4ZX85					GGDEF	EAL
D4ZXA5		H1WID7	UPI00061B2666		GGDEF	EAL
D4ZZM1		H1WJ37		B5VW83	GGDEF	EAL
D5A1Y8	K1W2J8	H1WAE6	UPI00061B0B37	B5W9L6	GGDEF	EAL
D5A284	K1W3W0	H1WAY4	UPI00061B4ED1	B5W2N1	GGDEF	EAL
D5A3S6		H1WBU5	UPI00061AE096	B5W5T2	GGDEF	EAL
	K1W281	H1W679	UPI00061A8FBA	B5VVW5	GGDEF	EAL
		H1WIC2			GGDEF	EAL
D5A623					CGDEF	EAL
D4ZRB6	K1WP85	H1WLU6	UPI00061B1286	B5VUP3	GADEF	EAL
D4ZRU3				B5VU37	TSGEF	EAL
D4ZP21					MADEF	ESL
		H1WLM6		B5VU82		Degenerate
D4ZP22	K1WN45			B5VYX9		EAL
D4ZS04						

D4ZVR0	K1WIL1			B5VXA8	
			UPI00061B1576	B5VVX1	
D5A586	K1W350	H1W7C7	UPI00061B31A7	B5W613	HD-GYP
D4ZQ27		H1WKU5			Degenerate
D4ZS60				B5W2D8	HD-GYP
D4ZUJ2		H1WBA5			
D4ZWS7	K1WA74	H1WK65		B5W225	
D4ZXH7					
D5A1B9		H1WHF4	UPI00061A98A7	B5VWT7	
D5A5J2	K1XFW9	H1W732		B5VV09	

Table S2. Meta-analyses of *Arthrospira* transcriptomes under different conditions.

Condition	Strain	Sample	Treatment	Log_2(FC) *adc11*	Log_2(FC) *ahd1*
1	*A*. sp. PCC8005	GSE67839	Normal conditions versus nitrogen-limited conditions	-0.04	0.16
2	*A*. sp. PCC8005 P2	GSE175921	Chronic gamma radiation	-0.56	-0.24
3	*A*. sp. PCC8005 P1	GSE63250	gamma rays, 800gy, 2h	-0.24	0.08
4			gamma rays, 800gy, 5h	-0.50	-0.04
5			gamma rays, 1600gy, 2h	-0.15	0.19
6			gamma rays, 1600gy, 5h	-0.35	0.10
7			gamma rays, 3200gy, 2h	-0.083	0.19
8			gamma rays, 3200gy, 5h	-0.10	0.20
9	*A*. sp. PCC8005 P2	GSE63250	gamma rays, 800gy, 2h	-0.18	-0.06
10			gamma rays, 800gy, 5h	-0.43	-0.09
11			gamma rays, 1600gy, 2h	-0.16	0.11
12			gamma rays, 1600gy, 5h	-0.36	0.06
13			gamma rays, 3200gy, 2h	-0.04	0.13
14			gamma rays, 3200gy, 5h	-0.14	0.14
15	*A*. sp. PCC8005 P3	GSE63250	gamma rays, 800gy, 2h	-0.18	-0.04
16			gamma rays, 800gy, 5h	-0.12	-0.04
17			gamma rays, 1600gy, 2h	-0.47	-0.04
18			gamma rays, 1600gy, 5h	-0.14	0.02
19			gamma rays, 3200gy, 2h	-0.43	0.02
20			gamma rays, 3200gy, 5h	-0.23	0.10
21	*A*. sp. PCC8005	GSE57456	60Co gamma rays, 3200gy	-0.13	0
22			60Co gamma rays, 5000gy	0.24	0.04

Table S3. Primers used in this study.

Name	Sequence (5'-3')	Target
Pcat-F	AGAGTTTGTAGAAACGCACGCAACGCAATTAATGTGAGTTACC	*cat* promoter
Pcat-R	GAGAGCAAATTTACTAATTGTTATCCGCTCACAATTCCACA	
Tcat-F	TTTTTTTAAGGCAGTTATTGGTGCCCTTAA	*cat* terminator
Tcat-R	ATCCTGACGGATGGCCTTTTAGAAGCACACGGTCACACTGCTT	
VC1-F	AGTAAATTTGCTCTCGGTCACACT	VC1 riboswitch
VC1-R	CATTTTAGGTTGTTTTTTCATCACAGACTC	
lacZ-F	AAAAAACAACCTAAAATGACCATGATTACGCCAAGCGCGCAA	*lacZ*
lacZ-R	ACTGCCTTAAAAAAACTAATCAAGTTTTTGGGGTCGAGGT	
pBad18-F	TGCGTTTCTACAAACTCTTTTGTTTA	pBad18 linearization
pBad18-R	AAAAGGCCATCCGTCAGGATGG	
*adc11*_D5A515-F*	CAGCAAATGGGTCGCATGAATGAGTTAATGGAAGACCGA	*adc11*_D5A515
*adc11*_D5A515-R	TCCGCCAAAACAGCCAATTGTCTCAACATTAGCAATGAC	
*adc11*_K1W5H6-F	CAGCAAATGGGTCGCATGAATCAGTTAATGGAAGACCGA	*adc11*_K1W5H6
*adc11*_K1W5H6-R**	TCCGCCAAAACAGCCAATGGTCTCAGCATTAGCAATTATGCA	
*adc11*_H1WDI7-F*	CAGCAAATGGGTCGCATGAATCAGTTAATGGAAGACCGA	*adc11*_ H1WDI7
*adc11*_H1WDI7-R**	TCCGCCAAAACAGCCAATGGTCTCAGCATTAGCAATTATGCA	
*adc11*_AD67D-F*	CAGCAAATGGGTCGCATGAATCAGTTAATGGAAGACCGA	*adc11*_AD67D
*adc11*_AD67D-R**	TCCGCCAAAACAGCCAATGGTCTCAGCATTAGCAATTATG	
*adc11*_B5VVN4-F*	CAGCAAATGGGTCGCATGAATCAGTTAATGGAAGACCGA	*adc11*_B5VVN4
*adc11*_B5VVN4-R**	TCCGCCAAAACAGCCAATGGTCTCAGCATTAGCAATTATGCA	
*ahd1*_D5A586-F	CAGCAAATGGGTCGCATGGTTTTTGATAATCTTAATGTGTCTG	*ahd1*_D5A586
*ahd1*_D5A586-R	TCCGCCAAAACAGCCAGCCAGTTCCATAACCACATCTAAA	
*ahd1*_K1W350-F	CAGCAAATGGGTCGCATGGTCTTTGATAATCTTAATGTGTCTG	*ahd1*_K1W350
*ahd1*_K1W350-R	TCCGCCAAAACAGCCAGCCAGTTCCATCACCACATCTAAA	
pBad-VC1-*lacZ*-F	GGCTGTTTTGGCGGATGAGAGAAGATTTT	Linearization of pBad-VC1-*lacZ*
pBad-VC1-*lacZ*-R	GCGACCCATTTGCTGTCCACCAGTCAT	

* represent identical upstream primers of *adc11*; ** represent identical downstream primers of *adc11*.

Table S4. Strains and plasmids used in this study.

Strain	Plasmid	Genotype
ΔVC1-lacZ	pBad-VC1-*lacZ*	KanR, VC1 riboswitch, *lacZ*
ΔD5A515	pVC1-lacZ_ΔAdc11_D5A515	KanR, VC1 riboswitch, *lacZ*, *adc11*_D5A515
ΔK1W5H6	pVC1-lacZ_ΔAdc11_K1W5H6	KanR, VC1 riboswitch, *lacZ*, *adc11*_K1W5H6
ΔH1WDI7	pVC1-lacZ_ΔAdc11_H1WDI7	KanR, VC1 riboswitch, *lacZ*, *adc11*_ H1WDI7
ΔUPI00061AD67D	pVC1-lacZ_ΔAdc11_AD67D	KanR, VC1 riboswitch, *lacZ*, *adc11*_UPI00061AD67D
ΔB5VVN4	pVC1-lacZ_ΔAdc11_B5VVN4	KanR, VC1 riboswitch, *lacZ*, *adc11*_B5VVN4
ΔD5A586	pVC1-lacZ_ΔAhd1_D5A586	KanR, VC1 riboswitch, *lacZ*, *ahd1*_D5A586
ΔK1W350	pVC1-lacZ_ΔAhd1_K1W350	KanR, VC1 riboswitch, *lacZ*, *ahd1*_K1W350
